# Changes in the body weight of term infants, born in the tropics, during the first seven days of life

**DOI:** 10.1186/1471-2431-13-93

**Published:** 2013-06-14

**Authors:** Claudia Turner, Verena Carrara, Naw Aye Mya Thien, Naw Moo Ku Paw, Marcus Rijken, Rose McGready, François Nosten

**Affiliations:** 1 Shoklo Malaria Research Unit, Mae Sot 63110, Thailand; 2 Mahidol-Oxford Tropical Medicine Research Unit, Bangkok 10400, Thailand; 3 Centre for Tropical Medicine, University of Oxford, Oxford OX3 7LJ, United Kingdom

**Keywords:** Neonate, Weight loss, Weight gain, Weight velocity

## Abstract

**Background:**

Identifying unwell neonates, particularly in the first week of life, is often subjective. If normal values are known, calculating the weight lost or gained from birth weight can be a useful adjunct in the evaluation of the health of a neonate.

**Methods:**

Serial body weights of well, term, breast fed infants who were attending for routine follow up, were recorded at the Shoklo Malaria Research Unit clinic in Maela Camp for displaced persons on the Thailand Myanmar border. Newborn examination was routine. Weight loss, expressed as percent weight lost from birth weight, and weight gain, expressed as a velocity (g/kg/day), was calculated for the first seven days of life. The results from normal birth weight infants, low birth weight infants (<2.5 kg) and small for gestational age infants (SGA) were examined.

**Results:**

In the first week of life there were no significant differences in weight gained or lost across the three study groups. The maximum weight lost was 4.4% (95% CI 4.1 – 4.6%), which occurred on day three. Weight gain ranged from 13 g/kg/day [95% CI 10 – 16] on day four to 18 g/kg/day [95% CI 15 – 20] on days six and seven.

**Conclusions:**

Use of these normal values for weight gain and loss, allows infants falling outside of the expected range (95% CI) to be easily identified and subsequently highlighted as needing further medical review.

## Background

Each year four million neonates die, the majority in the first week of life [1]. Most deaths occur in the developing world and are due to neonatal sepsis, birth complications and prematurity [1,2]. To have any hope of achieving the 4th Millennium development goal (a two third reduction of deaths in children less than five years of age), a decrease in the number of neonatal deaths must occur [2]. Assessing a neonate for signs of illness is often subjective. An unwell neonate can have subtle signs which can be missed, even by trained health care workers. Infants with neonatal sepsis can present in a number of ways: respiratory distress, temperature instability, cyanosis, hypotension, lethargy, hyperbilirubinaemia, abdominal distension, prostration, weak or absent movement, abnormally irritable or sleepy, abnormal feeding, inability to console infant, or with a bulging fontanelle [3-5]. Therefore, an objective measurement of neonatal wellbeing would be an invaluable tool, especially in resource poor settings where other diagnostic capacities are limited.

It is well established that infants will lose weight in the first few days of life [6]. Studies performed in developed countries quote a weight loss of between 4 – 7% in the first days of life [7,8]. However, there is a paucity of data regarding weight loss in normal infants in developing countries.

Calculating weight loss or weight gain is useful in the evaluation of a neonate. For example a weight loss of greater than 10% has been associated with dehydration and hypernatremia [9]. As part of an initial assessment, calculating whether the infant has lost more weight than would be expected for their age can guide management by objectively demonstrating at risk infants (for example infants who have lost too much weight from insufficient feeding or increases loses from diarrhoea). Calculating weight gain and comparing to expected values can aid evaluation of an infant’s recovery. For these useful calculations to be made, the expected weight loss and weight gain must be known.

In conventional paediatric practice weight loss in the first seven days of life is described as a percentage loss from birth weight and weight gain as a growth velocity (g/kg/day) [10].

The aim of this analysis was to describe the normal weight change in a breast fed infant in the first seven days of life and to evaluate whether this change was the same for normal weight infants, low birth weight infants (< 2.50 kg) and small for gestational age infants.

## Method

Maela Camp for displaced persons is located in North West Thailand in the hills adjoining the Myanmar border, 60 km north of Mae Sot. Maela has a population of approximately 43,000 people, living in an area of 4 km^2^. The Shoklo Malaria Research Unit (SMRU) clinic provides all antenatal care in the camp, where 1,500 deliveries occur each year.

Delivery in the SMRU suite is encouraged and newborns are routinely weighed in the first 30 minutes of life. At SMRU gestation is routinely estimated either by USS at the first antenatal consultation or by Dubowitz gestational assessment [11,12]. After delivery at the SMRU clinic, all mothers were asked whether they would like to bring their infants back to the clinic daily for 3 – 7 days for a health check. This was a voluntary assessment and no data on women who refused were recorded. Only women who lived a short walking distance from the clinic accepted as all women had to come on foot. The mothers of infants who were admitted to the SMRU hospital, within the first seven days of life, were not asked to participate. Body weights were measured routinely in these infant assessments and were recorded in the infant’s health records. Breast fed term infants who attended follow up from January 2007 to October 2011 were identified from these records. Infant weight was measured on scales with a reported accuracy of 10 g (model 335; Seca Ltd, Birmingham, UK). Scales were calibrated weekly with standardised weights (500 g, 1 kg, 2 kg, 3 kg, 5 kg and 8 kg). An infant was determined to be small for gestational age (SGA) if their weight fell below the 10th centile for weight, using normal population values of weight for gestation [13].

### Calculation of weight loss and gain

Weight loss was calculated as a percentage of the weight lost from birth to the day measured:

[(birth weight – today’s weight) ÷ birth weight] × 100.

Growth velocity was calculated as the g per kg lost each day:

[(weight today – weight yesterday) ÷ weight yesterday] × 1000.

### Statistical analysis

Data were entered into an Excel 2003 spreadsheet (Microsoft, Redmond WA, USA) and statistical analyses were carried out using STATA 12.1 (StataCorp, College Station TX, USA) and GraphPad Prism 5.04 for Windows, (GraphPad Software, La Jolla CA, USA). Continuous variables were described by the mean, standard deviation (SD) and 95% confidence interval (CI). Student’s t-test was used to compare means, with two-tailed p-values of <0.05 indicating significance.

Infants who were low birth weight and small for gestational age were included in both groups for the analysis of weight loss and weight gain.

### Ethical approval

Approval for retrospective analysis of hospital records was granted by the Oxford Tropical Research Ethics Committee (Reference 28–09).

## Results

Records were identified for 466 term infants who were brought for a daily health review and weight check between January 2007 and October 2011 (Table 1).

**Table 1:** Number of weight observations according to postnatal age and birth weight category

**Age**	**Number of observations**	**Number of observations (% of all observations)**		
		**Normal birth weight**	**Low birth weight**	**SGA**
Birth (Day 1)	466	359 (77.0)	103 (22.1)	74 (15.9)
Day 2	462	359 (77.7)	103 (22.6)	74 (16.0)
Day 3	455	355 (78.0)	100 (22.0)	71 (15.6)
Day 4	194	107 (55.2)	87 (44.8)	58 (29.9)
Day 5	190	104 (53.6)	86 (45.3)	58 (30.5)
Day 6	190	104 (53.6)	86 (45.3)	58 (30.5)
Day 7	189	104 (55.0)	84 (44.4)	57 (30.2)

The mean gestation of the infants was 38.2 weeks (SD 1.2 weeks; range 37.0 – 42.6 weeks) and 241/466 (51.7%) of the infants were male. There was no difference in follow-up duration between girls and boys. Of all the infants included in this study the mean birth weight was 2.95 kg (SD 0.47 kg), 103/466 (22.1%) had a birth weight of less than 2.50 kg (low birth weight, LBW) and 74/466 (15.9%) were SGA. Of the SGA group 89.2% (66/74) were also LBW.

**Figure 1:**
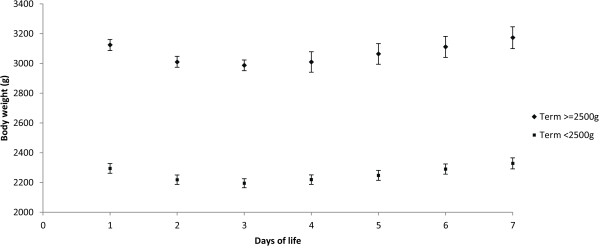
Mean body weight (95% CI of mean) from birth until day seven in breast fed, term, LBW and normal birth weight infants.

All infants (LBW, SGA and normal weight) lost weight until day three and subsequently gained weight, with birth weight being regained in all infants by day six (Figure 1).

### Weight loss

For all infants, the mean maximum weight loss was 4.4% (95% CI 4.1 – 4.6%) and this occurred on day three. For non LBW infants, the mean maximum weight loss was 4.5% (95% CI 4.2 – 4.8%) and for LBW term infants it was 4.1% (95% CI 3.5 – 4.7) (p = 0.3). A similar picture was seen in SGA infants with a 4.0% (95% CI 3.2 – 4.9%) weight loss on day three. There was no significant difference in weight loss, at any time point, between the sexes.

### Weight gain

Infants started to gain weight between days three and four with a mean gain of 13 g/kg/day (95% CI 9 – 17 g/kg/day). Weight gain subsequently continued until day seven (Table 2). There was no significant difference between the daily weight gain between normal weight, LBW and SGA infants.

**Table 2:** Mean weight gain (g/kg/day) by age (days) for all infants

**Days of age**	**Mean weight gain (g)**	**95% CI**
4	13	10 - 16
5	16	13 - 19
6	18	15 - 20
7	18	15 - 20

From day two until day six there was no significant difference between the weight gain velocity between boys and girls. However on day seven girls had a significantly greater weight gain than boys (20 g/kg/day vs. 15 g/kg/day, p = 0.04).

## Discussion

In this population, we have shown that a healthy term breast fed infant born in the tropics, loses no more than five per cent body weight after birth, with the maximal loss on day three, and regains birth weight by day six of life. Although one may speculate that, because of climate and possible increased insensible losses, there may be differences in the changes in body weight of an infant born in the tropics our results are similar to those previously reported in the literature [6,8].

The categories, LBW and SGA were included as in many parts of the developing world accurate dating of a pregnancy is not feasible. Consequently determination of whether an infant is SGA is not possible and only a classification of LBW is possible. Our study demonstrated no difference between normal weight, low birth weight and SGA infants. This has not been previously reported, since studies looking at normal weight gain often exclude infants with LBW or who are SGA. This should be a useful guide for health workers.

After day three, infants gained weight, having a weight velocity of between 13 – 18 g/kg/day (95% CI 10 – 20 g/kg/day). The WHO published weight velocity standards report a median of 14 g/kg/day weight gain in girls and 21 g/kg/day gain in boys across all birth weights [14,15]. Although this data is based on infants from both the developed and developing world, the range is similar to our results; however we did not see a greater weight gain in boys compared to girls. This finding could be explained by the fact that parental favouring of one sex over another has not been reported in our population but is reported to be common in certain geographic locations [16].

This study is limited by the fact that it is an observational retrospective study in a small geographical location. Further research, on this important topic, is warranted in other locations in the developing world.

## Conclusion

Information on normal weight gain and weight loss in the first seven days of life has important clinical implications. It gives health workers a valuable adjunct in the assessment of the clinical state of an infant in resource poor settings where the capacity for alternative investigations is extremely limited.

## Competing interests

The authors report no potential conflicts of interest.

## Authors’ contributions

CT, VC, RM and FN conceived the study. AMT, MKP, MR and CT were responsible for data collection. CT did the data analysis and prepared the first draft of the manuscript. All authors reviewed and contributed to revisions of the manuscript. All authors read and approved the final manuscript.

## Pre-publication history

The pre-publication history for this paper can be accessed here:

http://www.biomedcentral.com/1471-2431/13/93/prepub
